# Time as a Key Topic in Health Professionals’ Perceptions of Clinical Handovers

**DOI:** 10.1177/2333393614550162

**Published:** 2014-10-16

**Authors:** Bernadette M. Watson, Liz Jones, Julia Cretchley

**Affiliations:** 1 The University of Queensland, Brisbane, Queensland, Australia; 2 Griffith University, Brisbane, Queensland, Australia

**Keywords:** communication, concept mapping, health care, culture of, health care, interprofessional, interviews, semistructured, quality of care, social identity

## Abstract

Clinical handovers are an essential part of the daily care and treatment of hospital patients. We invoked a language and social psychology lens to investigate how different health professional groups discussed the communication problems and strengths they experienced in handovers. We conducted in-depth interviews with three different health professional groups within a large metropolitan hospital. We used Leximancer text analytics software as a tool to analyze the data. Results showed that time was of concern to all groups in both similar and diverse ways. All professionals discussed time management, time pressures, and the difficulties of coordinating different handovers. Each professional group had its own unique perceptions and priorities about handovers. Our findings indicated that health professionals understood what was required for handover improvement but did not have the extra capacity to alter their current environment. Hospital management, with clinicians, need to implement handover schedule processes that prioritize interprofessional representation.

Clinical handovers are a key communication event involving “the transfer of professional responsibility and accountability for some or all aspects of care for a patient, or group of patients, to another person or professional group on a temporary or permanent basis” (Australian Commission on Safety and Quality in Health Care [ACSQHC], 2012; Australian Medical Association, 2006). Clinical handovers, however, along with other aspects of patient procedures, do not operate in a vacuum but rather form one part of health care in the busy and complex organizational structure of hospitals.

These complexities can adversely influence communication in hospitals, including the handover context. Sutcliffe, Lewton, and Rosenthal (2004) found that faulty communication was an associated or contributory factor in 91% of mishaps reported by medical residents. Information about patients was often seriously or completely lacking. Sutcliffe et al. concluded that failures of communication are not simply the result of faulty information transmission but reflect professional hierarchical differences involving concerns with upward influence, interpersonal power and conflict and role ambiguity.

In this article, we focus on communication in the handover context because it is the interface of much of a patient’s care. There are many types of handovers involving different groups of health professionals. However, often the term handover has been used as a blanket term to cover a diverse range of exchanges between clinicians about ongoing patient care. We argue that the diversity of handovers, the communication styles of different health professions, and the hospital as a hierarchical hospital system are contextual factors that influence the communication process.

## Issues Around Effective Communication During Clinical Handovers

Although research into clinical handovers is growing, up until the mid-2000s it was not a well-researched area (ACSQHC, 2005). Recent research identifies a range of concerns about the effectiveness of clinical handovers that implicate communication. Solet, Norvell, Rutan, and Frankel (2005) noted four communication barriers to effective handovers. Of importance to this article is their finding that status differentials between health professionals impeded open discussion and clarification requests.

The status differentials among professionals raised by Sutcliffe et al. (2004) and Solet et al. (2005) negatively affect effective communication and highlight that hospitals are intergroup institutions with professions possessing their own cultural ingroups. Researchers have shown that communication problems are exacerbated where professions or specialties have subcultures with different core values (Epstein, 1995; Grice, Gallois, Jones, Paulsen, & Callan, 2006; Hewett, Watson, Gallois, Ward, & Leggett, 2009). These cultural differences are evident when comparing the different handover practices between medicine and nursing.

Leonard, Graham, and Bonacum (2004) noted that nurses use a broad and narrative approach, which includes each patient’s daily routine details such as fluid intake and bowel movements. In contrast, doctors tend to use a more concise approach that focuses on patient symptoms and treatment. They often use the acronym SOAP (Subjective assessment of patient, Objective assessment of patient, Assessment of patient, and Plan for patient) to guide their evaluation. These differences in patient reporting might be problematic when doctors and nurses attend the same handover. Mitchell, Parker, Giles, and Boyle (2014) found that the diversity of interprofessional teams can lead to dysfunctional relations because each profession emphasizes a different patient focus.

Most research into clinical handovers is conducted by researchers outside the hospital system, who most often observe handovers or run large surveys that investigate communication skills. The outcomes from these studies are generally recommendations for a change in tools or procedures to improve handovers and hence patient care (see Wong, Yee, & Turner, 2008). Wong and colleague’s (2008) report noted that, despite increased attention to handovers, changes in practice were not evident. It also noted the “complex and dynamic nature of handovers” (p. 3) and their diversity. Standard 6 of the National Safety and Quality Health Service (NSQHS) Standards (ACSQHC, 2012) provides a clinical handover matrix that articulates seven situations when handover should occur and nine delivery modes. This matrix reflects the diversity of handovers. With the increased focus on interprofessional practice, handovers also increasingly involve more than one health professional group.

In the current study, we asked interdisciplinary clinicians working in the hospital culture to tell us their experiences. We explored how different health professional groups articulate the handover process. By asking clinicians from different professions to discuss key problems and strengths across different handover situations, we aimed to illuminate how clinicians understand and negotiate different clinical handover contexts. We deliberately focused on one area of practice (an internal medicine facility at a tertiary hospital) to ensure consistency of clinical contexts across health professions and their experiences of handovers.

We invoked a language and social psychology (LSP) framework to investigate how different health professionals described diverse types of handovers. A LSP approach emphasizes that an interactant’s motivations and cognitions about a given encounter influence his or her perceptions about that event, which in turn influences his or her current and subsequent behaviors during similar events. It is well suited to investigating health professionals’ perceptions of handovers and their responses to them because it acknowledges that different health professional disciplines have different values and beliefs (cf. Epstein, 1995; Grice et al., 2006; Hewett et al., 2009; Mitchell et al., 2014). Embedded in a LSP perspective is the recognition that individuals bring their own values, beliefs, and previous relevant experiences to each and every interaction. This background influences how participants negotiate their roles with other health professional disciplines.

In the health context, there is extensive research showing how interactions between different health professions or subspecialties might be intergroup. Stein (1967) noted “the doctor-nurse game” with the status differentials between nurses and doctors. More recently, Lingard and her colleagues observed serious tensions in hospital teams and the presence of an “us” and “them” mentality that impeded working relations and collaboration (Lingard et al., 2005; Lingard, Reznick, DeVito, & Espin, 2002; Lingard, Reznick, Espin, Regehr, & DeVito, 2002). Using a LSP approach, Hewett et al. (2009) found intergroup tensions between doctors from different subspecialties that adversely affected patient care. Their article supported findings by Fewster-Thuente and Velsor-Friedrich (2008) that the intergroup dynamics of the hospital culture were a barrier to good health professional relations. These intergroup relations reflected a silo mentality that existed between professions and hindered good patient care (Kreindler, Dowd, Star, & Gottschalk, 2012)

In summary, clinical handovers are diverse and complex. Improving clinical handover communication requires an investigation of these complexities. We adopted a LSP lens to explore how health clinicians describe and manage clinical handovers. Specifically, we wanted to investigate how the cognitions (perceptions) of health professional groups differ regarding what makes handovers effective or ineffective. Given that handovers vary in their composition, we also wanted to know whether different health care professions had differing perceptions about the various types of handovers they experienced. By tapping into the knowledge and experience of different health professionals, new information and directions may emerge to improve clinical handovers. Our aim was to get in-depth data from a small representative sample of different professions to explore differences between professions who work in the same environment. Specifically, we asked the following:

**Research Question 1:** What factors do health care professions emphasize as enablers or barriers to effective and ineffective handovers?
**Research Question 2:** How do different health care professions differ in their perceptions of effective and ineffective handovers?

## Method

### The Facility

The study was conducted in the medical division of a large public tertiary hospital in southeast Queensland. The division covers a wide range of patient care including respiratory and vascular care, geriatrics, rehabilitation, and internal medicine. One of the challenges of studying clinical handovers across professional groups is the multiplicity of handovers which occur. This division had four main types of verbal handovers: nursing handovers between shifts (which in this division included both face-to-face handovers and taped—the latter occurring before a shift ended and the next one commenced so the nurses did not see each other), daily post-take rounds, where the night doctor provided information to a consultant and the doctors on the incoming day shift about the patients who had been admitted overnight. This handover occurred in small areas away from the patients’ beds. On completion, the night doctor left and the consultant and day shift doctors visited each patient in turn. At this point, allied health professionals and relevant nurses, when available, could join the ward round.

The third and fourth types of handovers were the case conferences and white board meetings between doctors, nurses, and allied health professionals. Typically, case conferences occurred in a meeting room, whereas the white board meetings were brief and occurred by the white board at the nurses’ station. Owing to the similarity in terms of interprofessional mix between these last two handovers, we term these interprofessional handovers and discuss them together. At interprofessional handovers, all health professionals involved in a patient’s care were expected to attend to discuss patient management. Health professionals often attended more than one type of handover, and these handovers were either intragroup (only one health profession involved) or intergroup (more than one health profession involved).

### Participants

Twenty-two participants who volunteered were interviewed for the study. The sample included 9 medical practitioners, 9 nurses, and 4 allied health professionals (2 social workers, 1 occupational therapist, and 1 physical therapist). The smaller number of allied health professionals is proportionate to their representation in the medical division of the hospital. The participants were representative of both senior and junior health professionals, who had worked at the hospital for between 1 and 30 years. The participants were recruited from different wards but all worked in the medical division of the hospital. For the participant identification, we gave each participant a number that signified the order in which the interview occurred and added the participant’s profession.

### Procedure

Ethical approval was obtained through Queensland Health and the University of Queensland (2006000979). The director of internal medicine sent a letter to all staff in the medical division outlining the purpose of the study and asking for participants. The researchers then contacted the nurse unit managers (NUMs) and the doctors in the medical division to request volunteers. We used a purposive sampling technique to recruit a representative sample of doctors, nurses, and allied health staff. Prior to commencing the interview, participants were given an information sheet regarding the project, which included an assurance of their anonymity. Participants then signed a consent form. The participants answered a series of open-ended questions, which included asking them what they saw as “effective” and “ineffective” aspects of handovers. We also asked participants what, if anything, they would like to see changed, giving participants an opportunity to discuss what they would do to alter the current handover process. Our final question asked was “What is the most important aspect of a clinical handover?” Participants were given the opportunity to raise and discuss points beyond the predetermined questions. Interviews lasted on average 35 min and ended when the participants had fully answered the questions. The interviews were digitally recorded and transcribed verbatim.

### Leximancer Analysis

We used Leximancer text analytics software, version 4, to analyze the interview texts. Leximancer is a computer program that uses word association information to allow concepts to emerge automatically from electronic text (Smith, 2003). In Leximancer, a “concept” is a list of related words which participants use together often (and seldom apart) in the data (Leximancer, 2011). It works to analyze text in this way because the words people use together (within a tight, two-sentence space) are usually related to one another somehow. They tend to express some core idea, and these ideas can be identified by considering patterns of word usage across the text.

Using co-occurrence statistics to discover emergent concepts allowed us to avoid applying any predetermined rules or using a general dictionary (Smith & Humphreys, 2006). This reduced the risk that the code frame was not relevant to the data or omitted crucial components. It meant that we did not adopt a particular theoretical approach to data analysis. Some implicit references could be captured, for example where a keyword was absent but sufficient related terms existed within the text block to suggest a concept. This process is superior to keyword searching, and is more like human coding where the analyst reads into the text.

There are some limitations to Leximancer’s method, and the most important of these is that the approach is quite literal. It relies on the actual words used in the interview transcripts, therefore most (but not all) of the emergent concepts are nouns. The results naturally differ from those of manual coding, though, of course, human coding is vulnerable to issues of reliability and validity. Not all implicit references are handled as they would be if a human reading was applying theory to the data, and some complex and nuanced references might be missed. Leximancer also identifies what is present in the data, without highlighting what is absent (as might be possible if theory was being applied).The Leximancer results thus only take the analysis so far. The researcher must then take over to perform quality control and to interpret the findings. The first two authors read the transcripts multiple times to check the findings and to validate the accuracy and meaning of the two Leximancer analyses.

When a set of concepts has been identified and coded into the text, Leximancer presents these results using a two-dimensional map using clustered co-occurrence information. Those mentioned together often attract one another strongly, and so tend to lie near one another. The map can therefore be interpreted by considering the relative positions of concepts. In this case, each transcript was labeled according to the participant’s profession (nurse, doctor, or allied health professional), and these file names were included as labels on the maps. This allowed us to compare the concepts associated with the different health disciplines.

## Results

We created an exploratory map of all the transcripts. We included the labels representing each professional group on the map to facilitate a comparison between professional groups examining whether any themes occurred that were common across all the health professionals interviewed, and how these related to their perceptions of enablers of and barriers to effective and ineffective handovers (Research Question 1). We then produced a difference analysis map that extracted themes that were unique to each professional group. This analysis revealed one separate unique concept for each professional group (Research Question 2). The maps created are linked with the text that created the concepts allowing researchers to link what participants said with the relevant concepts.

### Exploratory Analysis Map

The exploratory Leximancer map contained many concepts which described the diversity of handover experiences for the professions, but the layout revealed one major theme (cluster of concepts) identifying a key issue for all professions. This theme is shown in the large circle on the map, and centered on the concept of “time.” Issues to do with time were discussed the most by all participants, so the following results focus on how time influenced handovers. Each of the other (less important) themes on the map lay near one of the professional groups, suggesting differences in the foci of their comments. These differences are discussed later in the results, where we performed a difference analysis.

First, we discuss how “time” was a key issue for each of the health professional groups. All professionals referred to how the daily timing of handovers affected their ability to attend particular types of handovers. Doctors stated that they would like the nurses to accompany them because the nursing perspective was important and improved the effectiveness of the handover. One doctor commented,

Clearly, if there’s been any observations made by nursing staff or others since the patient’s been in hospital that they think are important for us to know . . . that’s why we like to have nurses accompany us on the post-take rounds so that they can tell us what they’ve observed over the time that they’ve been with the patient.

However, they reported that there were time constraints as to who could attend their post-take ward round. Specifically, doctors acknowledged that the timing of their post-take ward was when nurses were most occupied with patient care. Nurses also reported that they would like to participate in the doctors’ post-take ward round but that the timing of the round made it impossible because it clashed with busy times on the ward:

But we have had problems [with timing] because we also need the nurse looking after the patient to attend the post-take round for the patient and it’s usually falling when they’re doing hygiene cares or on their tea breaks or things like that so they don’t often attend.

Another nurse stated, “Negative wise, the nurses often aren’t notified that the [post-take] round is happening . . . They [post-take ward rounds] often occur in the peak, busy time for nursing, ADL [assisted daily living] type care.” One nurse suggested that a mutually convenient time could be negotiated “so an established time maybe, an approximate, established time, for the medical staff to actually notify the nurse in the area that they’re performing the post-take.”

With respect to interprofessional handovers (white board or case conferences), doctors again stressed the importance of having other health professionals attending. This doctor spoke about case conferences:

I mean, the big advantage with that is having multiple allied health teams at case conferences so that everyone can discuss everyone all at once otherwise you’re trying to talk to four or five different teams and it’s quite disorganized . . . from the point of view of the different allied health teams everyone bounces ideas off each other so it’s more integrated.

Allied health professionals also spoke about the importance of discussing patient care with other health professions. In line with other professions, they also described the challenge of accessing the correct health representatives to discuss patient status. They discussed how it had to be the appropriate representative, who knew about the patients. They reported that they had difficulty finding a nurse who would be able to inform them about a patient’s condition. For example,

It’s very difficult to find a nurse who can . . . every time you ask a nurse how is this patient going? “Well, we’ve only been looking after this patient for two hours, they will say . . . we have no idea.”

Another allied health professional said that it was unhelpful to have constantly different health professionals at meetings for the same patients because there was little continuity of care. “So if there was a different OT [occupational therapist], say, or a different doctor or a different nurse in charge then you’re not getting as much as the continuity sometimes, if things aren’t recorded.”

Our findings suggest a lack of timing coordination between the different types of handovers. For example, one doctor commented that one of the interprofessional handovers was poorly timed because he or she was on the post-take ward round:

Timing, if it were done maybe with consulting—I think someone’s made a decision that perhaps the nurses have said, “Well, this is the best time for us” without asking anyone else . . . I don’t feel it’s the best time because the team decisions regarding the nature of ongoing care for a patient are really made during and after the ward round.

These results indicate that, at least in this internal medical division, better timing of the different handovers might improve the quality of information shared. There was agreement that nonattendance of the right “people” (professional knowledge) meant that staff could not share information about patients, which influenced handover effectiveness. The interprofessional handovers and the post-take ward rounds were targeted as ones where coordination of timing might improve representation. These comments all focused on handover improvements and did not overtly describe where “time” works well. It is implicit from their comments that enablers to good handovers are where there is correct staff representation and barriers are where there is not good representation.

**Figure 1.**
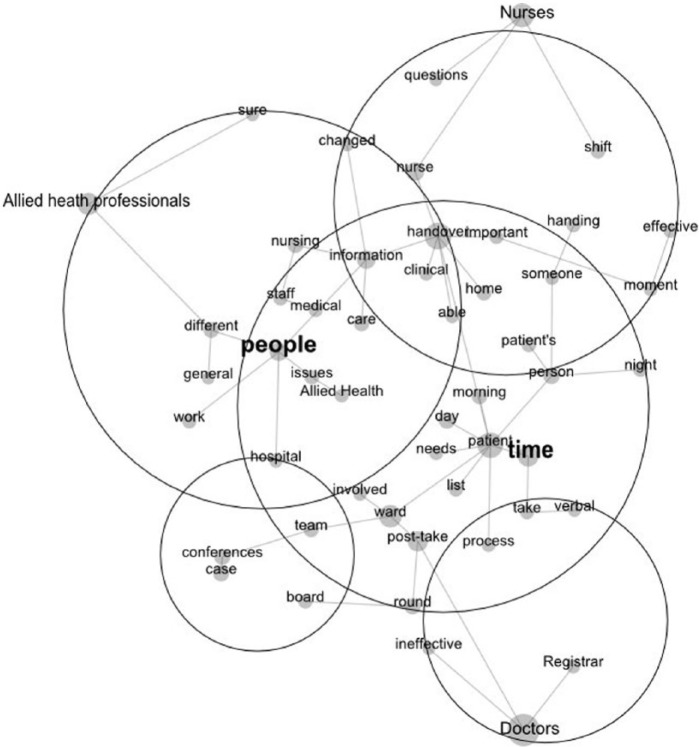
The Leximancer exploratory map of themes, with those discussed in the text enlarged and highlighted.

The different concepts clustered near each of the professional groups in Figure 1 showed that doctors, nurses, and allied health professionals possessed a unique focus on what “time” meant for them. To illustrate the differences, we used Leximancer to find exemplar quotes from participants that reflected the meanings of “time” to particular health professional groups and the importance of time.

Nurses specifically referred to time in terms of the quality of information covered at handovers. The comments ranged from superfluous information being shared to the suggestion that a time constraint be imposed on each patient’s handover information:

I think the most inefficient thing of the handover is people from the list repeating their history and what is wrong with them especially if they’re long term patients. We know what’s wrong with them . . . And that is hard for agency [nurses] because they’ve never been here so they need to know but they also then hand that over but, yeah, that’s just generally what we don’t want to hear.

One nurse noted that it might be about training nurses to prepare appropriately for handover: “I think it’s about educating people not to waffle.” Another nurse stated that written information does not need to be included in the verbal handover: “What works for me? If people tell me actually what’s happening rather than babble on about, you know, things that are already written in front of you.” This last quote demonstrates the tension between spending enough, but not too much, time discussing a patient. In contrast, the following nurse mentioned the disadvantage of time constraints: “Yeah, and sometimes four minutes [set time for each patient] seems like not enough time so you can miss things . . . But it is good, it does make people compact what they’re going to say.”

Doctors referred to the quality of information with respect to the format of presentation. They spoke about the medical division having introduced a template to improve handover information. The introduction of this system appeared to be an enabler to effective handovers. For example, the following doctor described the template as an important tool for facilitating a quick and effective handover:

Ineffective handover is where, I think, there’s absence of key information. So people not using a structured template, kind of relying more on memory or a few scribbled notes . . . And that’s really why we’ve brought the template in because I got sick of registrars speeding things and not knowing bits and pieces about their patients.

Doctors did, however, also note that time pressures, and their associated workload, prevented them from using these templates. Not having structured notes about patients at handover meant that they were inadequately prepared. One doctor spoke of having too many tasks to perform simultaneously and that the existing template was not always filled in. Supporting this comment, a doctor stated that there was just not time to write things down:

I think the main issue is that the registrar have got a lot of work overnight so ideally you’d want something, I guess, written down very brief but practically that’s not feasible since they’re trying to admit so many people at the same time.

When allied health professionals spoke about time they stressed that they needed more time because they had many wards to visit for handovers (one allied health professional spoke about working on 11 wards). The fact that allied health professionals were constantly trying to find time to participate in a large number of handovers suggests that for them it was access to handovers and not length of time in, or preparation for, handover that was important. For them, it was critical that they spoke to the correct representatives from the different health professions:

Because it’s just too many wards. It’d be good to go to the main ones, and I try and make it to the main wards that I see, but it’s just the time factor . . . If I went to all of them, or even if I went to a few of them, they would take out a lot [of] the morning because they never seem to run exactly on time.

This comment links back to their problem described earlier of not being able to access the relevant health professionals for patient information. Allied health professionals are the one group who do not do shift work. Given their high patient load, it is also relevant that they have to manage their schedules within their 8-hr day shift. This difference in working times is a cultural difference between allied health professionals and the other two groups, which might in turn become an organizational barrier in the work relationships between allied health and doctors and nurses.

These quotes demonstrate that all the health professionals were highly conscious of the difficulties of managing their time in their specific roles. The quotes emphasize that health professionals are “time poor” and this negatively affects handover effectiveness.

### Discriminant Analysis Map

**Figure 2.**
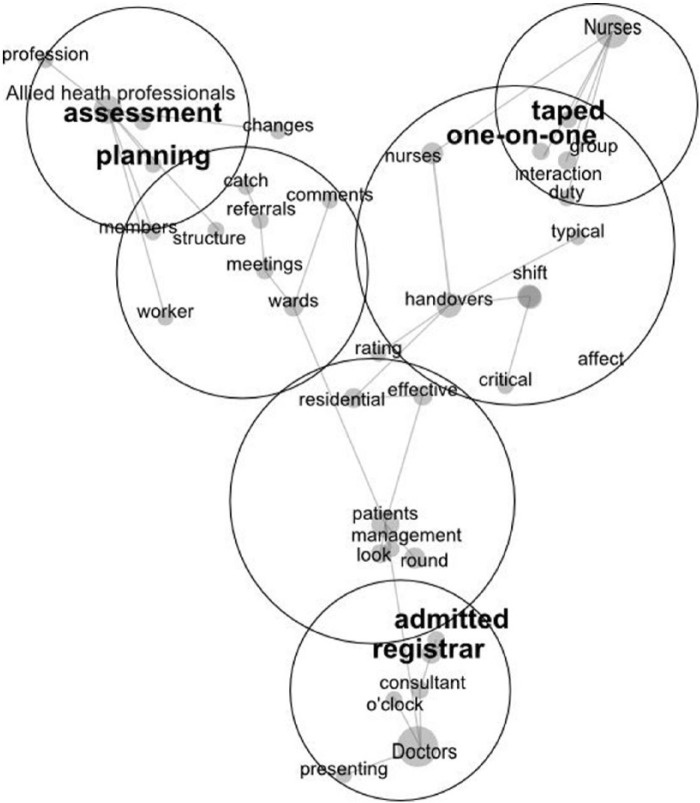
The Leximancer discriminant map of themes, with those discussed in the text enlarged and highlighted.

Each professional group also had unique concepts that did not cross to the other professions. We used Leximancer to examine the same interview texts again, but this time we asked for the concepts discovered to highlight differences between the professional groups (rather than identifying consensus issues). We included the label representing each of the professional groups on the map to facilitate a comparison. The concepts lying nearest each profession’s label on the map are those mentioned more by them than the other professions (see Figure 2).

The differences that emerged revealed how each of the three professions perceived different aspects of the handover as critical to them, which reflected their different roles. The map shows that doctors concentrated on concepts (“registrar” and “admitted”) that were about how registrars play a central role for doctors in their daily work of admitting patients. When we drilled into these two concepts, we found that the unique focus centered on how doctors were constantly trying to organize time to meet with registrars to discuss information about patients who had been admitted:

The night medical registrar needs to be updated upon any of the jobs that might remain incomplete at the time of admission, or any of the issues which might require medical attention overnight. Then again, it needs time where all the three [doctors] can sit and share between each other, what are the concerns which the admitted patient might require. We do that at this moment but I think we require a much more effective way of doing that.

This doctor suggested that to be effective they had to ensure that the medical staff were able to see each other during the working day and not just at a formal handover:

So that is the second aspect, that the morning after should be something like—either there should be a meeting before the post-take rounds where you can—say 15 to 20 minutes where all the registrars sit and all the consultants sit and say what were the admissions and what were the issues which need to be sorted, case by case. So that would be a handover plus a teaching type of thing. It can be more effective. That’s what I feel.

The transfer of information about patients continues after the official handover:

In the afternoon what usually happens is, if you’ve got a sick patient, you’ll just ring up the medical registrar on for the evening and I don’t think there needs to be any particular time for that, just kind of when you’re finished just to let them know about it.

This comment reinforces the fact that doctors were constantly actively managing different patients whose status changed during the day, and the post-take ward round was only one aspect of their constant sharing of patient information. In fact, the impression that emerged was that doctors found it difficult to coordinate their time with registrars within and across their shifts.

Nurses did not discuss ward admittance. Instead, the “taped” concept lay near the nurse’s label on the map, indicating that nurses were the only group to speak about taped handovers, which is not surprising because only nurses used this system in this hospital. However, the fact that it showed up as a strong difference between the nurses and other professions highlights that Leximancer successfully distinguishes differences in topics across groups. There were different viewpoints among nurses about the effectiveness of recording handovers:

You miss some of the personalities of the person’s interpretation on that by their physical stance and their interaction and that sort of stuff. So—and they may be a little bit guarded when they’re doing a tape handover.

And another nurse said,

I don’t believe in taped handovers because I think it’s at a disadvantage because . . . I’ve had to use taped handovers before and I think people get a bit stressed when they’ve got to talk into something so they can tend to forget their information or they might have to spend a bit of extra time writing it down to make sure that they get all the information across.

These comments also picked up on the importance of “one-on-one” encounters in handovers, which refers to face-to-face interactions and is missing with a taped handover. The problem of missing information was also noted when nurses spoke about taped handovers: “. . . we did taped handovers over there [another hospital], which is good as well but, yeah, you can miss information.” Although the unique focus for nurses was about taped handovers, it is clear that they, like the doctors, recognized the need to discuss patient care with professionals interpersonally.

An examination of the map revealed that allied health professionals spoke about patient “assessment” and “planning.” The allied health professionals used the handover as a time to assess how well the patients were rehabilitating and whether a plan could be commenced that led to timely discharge: “It just allows me to plan my workload as well as continue the care. If I don’t know the plan, if I have to do the initial assessment again and revise this, my planning could be delayed.” Another allied health professional said, “I think this is part of the social work role. That’s how I have seen it in gathering this information together and then we come out with an assessment about what is happening here.”

The allied health professionals’ focus differed from the doctors’ attention on treatment and timely care because it concerned future planning about the patient and assessment with an emphasis on patient self-management after discharge. Their presence at the interprofessional handovers reflected the different nature of these handovers because they focused on the future of the patient beyond immediate medical treatment and care.

The discriminant analysis demonstrated the different foci that each professional group had with respect to patient care. However, there were also commonalities across the three different topics (registrar/admitted, taped, assessment/planning) namely, trying to provide appropriate and timely patient treatment and management. Although this fact is not surprising, the fact that health professionals did focus on different aspects of patient care is not often considered as part of the hospital environment.

## Discussion and Practical Implications

We investigated the perceptions of different health disciplines about their experiences of effective and ineffective handovers. As noted in the introduction, many of the studies of handovers have adopted methods such as observation techniques or large survey designs, with the subsequent result that they recommend new processes and techniques to improve the handover (see Wong et al., 2008). These studies are valuable because they provide techniques and tools that help health professionals improve the handover process. However, they are insufficient because handovers do not operate in isolation but rather exist within the larger hospital system. This study looked at the bigger picture of handover procedures across different types or handovers with a range of health professional groups.

As interdisciplinary health care becomes the norm, it is timely to understand different professions’ understanding of patient care. We used a LSP approach to analyze handovers. LSP takes account of an individual’s cognitions and motivations in a given context and encounter that allows us to explain what health professionals perceive as important in managing handovers. Contrary to expectations, we did not find overt evidence of intergroup conflict between the three professional groups. Our findings suggest that across all these groups, time was a major focus of concern. Time was represented by the three groups in both similar and diverse ways. They all discussed time management, time pressures, and the difficulties of coordinating the business of handovers.

As the focus on interprofessional practice increases (Mitchell et al., 2014), our results show that the professions want different multidisciplinary representation at handovers. Such representation facilitates sharing a broader range of information about the patients than currently occurs. The three professional groups voiced frustration that the right representation of health professionals is not present at many handovers. They stated that the current handover structure is superimposed on them by the hospital system and is not well coordinated, which adversely affects handover quality. The related heavy workloads and time pressures of all health professions (in our sample) meant that they had no spare capacity to address these problems of handover scheduling and structure. All professions wanted to share their expertise at different handovers, although nurses were the one profession that did not suggest sharing nurse handovers with other health disciplines.

We drilled specifically into the dimensions of what is problematic about time for different health professions. Doctors and nurses perceived that the timing of handovers (post-take ward round and interprofessional) was problematic and could be better managed to be more inclusive of other professions and so make use of their collective knowledge to increase handover effectiveness. This finding suggests that at least some professions do not want to work in silos and would like a more shared approach to patient care. It also suggests that some health professions acknowledge the shortcoming of the current system and recognize the benefits of change.

There was, however, little evidence of different professional groups adapting their work schedules to accommodate this gap. So even though intergroup conflicts were not evident, there still appears evidence of a silo mentality and a strong intergroup dynamic (Epstein, 1995; Grice et al., 2006; Hewett et al., 2009). This might in part reflect a lack of leadership by more senior health professionals to oversee this process or the time pressure health professionals are under to perform other patient tasks. It might also reflect limited hospital managerial support or little realization that rescheduling of handovers can improve handover effectiveness. Whatever the reasons, it is time to bring health professionals and management together to devise solutions across the wider hospital system and demonstrate leadership across all health providers. Currently, much of the work to improve handovers focuses on within professions, and different solutions will be required for handovers that involve multiple professions. Yee, Wong, and Turner (2009) examined nursing and medical handovers but more interdisciplinary work is required.

Nurses did not suggest other professions attend their nursing handovers. This might reflect the nature of the nursing profession and their tradition of “nurses only” at handover (Manias & Street, 2000; Strange, 1996). Moreover, nurses, as frontline staff, provide the majority of patient day-to-day care, and the handover is an opportunity to discuss this care. The status differential between doctors and nurses can also explain the latter’s unwillingness to include doctors in their handovers. Changing this tradition might not be something that has been considered. However, further research is needed to identify why (and if) nurse only handovers are required or preferred. In contrast, nurses are keen to be part of the post-take ward round and doctors seem to welcome this.

Doctors and nurses described other issues related to the concept of time. Although there are differences across these two professions, both groups discussed the importance of adequate preparation and what should be the format of handovers. Ensuring that the necessary information is covered was regarded as key to effective handovers.

For nurses, the length of the handovers was an important issue, with ineffective handovers taking too long or conversely unrealistic time constraints for reporting a patient’s status. They expressed concern about handover content not being relevant, with superficial information being covered. This finding suggests that although the nursing handover system is the most traditional and ritualistic (Strange, 1996), there are still problems with getting the right balance of content and structure. Doctors did not talk about handover length but rather the importance of not rushing. This comment reflects the fact that doctors in this sample had a heavy workload resulting in a frenetic work pace. Even though doctors stated that a template to record the patient’s status was useful, time constraints meant that a template was not practical because they were too rushed to write down patient details. So although nurses and doctors raised different issues because of their different approaches and focus (Ilan et al., 2012; Leonard et al., 2004), they were both concerned with information presentation.

In contrast, allied health professionals reported constantly seeking out information from others (often nurses), so that they could establish the current state of a patient. The impression formed from these professionals was one of frantic seeking of information and traversing the hospital wards to find appropriate nursing staff. They spoke about the difficulties of locating people across different wards and the time it took to accomplish this. Part of this focus is brought about by the fact that allied health professionals are only in the hospital during the day (rather than over the 24-hr period) and are required to see a range of patients across a number of wards. It was apparent from their comments, that their daytime only time work schedule affected their ability to both manage their patient load and to obtain necessary information for optimal patient care.

In this article, we have demonstrated how these groups all have patient care at the center of their day but manage that care very differently. Our findings also support the fact that the three professions have their own specific views about handovers that reflects their different roles or experiences in the hospital system. For example, doctors described sharing patient care information with registrars and consultants across their working day, with the handover reflecting just one aspect of these clinical interactions to negotiate the management of patient treatment with others. McCann, McHardy, and Child (2007) noted that doctors’ handovers were much less structured than nurses at the same hospital.

In our study, this finding is reflected in the doctors’ less structured information sharing about patient care. Allied health professionals, in contrast, appear to be driven by planning and patient assessment with a view to discharge and rehabilitation, which reflects their clinical role. Nurses were differentiated from other health professionals by their discussion about taped handovers and the mechanics of what was detrimental to good handover practice. Indeed the Standard 6 of the NSQHS Standards (ACSQHC, 2012) now recognizes that taped handovers do not provide appropriate channels of communication.

This study was limited to health professionals in one division at one hospital; thus, we cannot generalize our finding to other hospitals or other facilities. That said it was also a strength, because having a restricted scope kept consistency in the clinical context across the interviews. A next step would be to investigate other divisions and other hospitals. We suggest that interprofessional focus groups around the handover process might provide in-depth insight into how professional groups can better manage handovers involving different professions. We also think that it is time for senior management to be brought into discussions about handover scheduling across professions.

The perspectives raised by these three professional groups, and displayed in the exploratory and discriminatory maps, demonstrate how “time” is a multifaceted concept and provide a bigger picture of the issues facing health professionals. What emerges is a picture of health professionals who do not have the capacity to change features of handover that might improve the process. This finding goes beyond the introduction of communication procedures and tools that can enhance communication skills. Although such changes improve the mechanics of handovers, they cannot be adopted to their full advantage because the hospital system in which handovers are embedded needs reviewing.

## Conclusions and Future Directions

Health professions have good insights into handover improvement. Hospital management, with clinicians, need to lead this process, and implement handover scheduling protocols that enable handover coordination that prioritizes interprofessional representation. This change might also reduce the current silo mentality that has been more generally observed across hospitals globally (Kreindler et al., 2012). There is no one solution or technique that can fix the problems raised. Changes to handover scheduling will affect the systemic hospital structure and require implementers to take a holistic view of the hospital structure, the range of health professions who manage handovers, and the different handover procedures. We need more studies to examine the different health professions’ perspectives and revisit issues identified in this study. We urge scholars to explore more closely how the different disciplines perceive their working day and how this perception shapes and motivates their work.
